# Neurobehavioral and Antioxidant Effects of Ethanolic Extract of Yellow Propolis

**DOI:** 10.1155/2016/2906953

**Published:** 2016-10-16

**Authors:** Cinthia Cristina Sousa de Menezes da Silveira, Luanna Melo Pereira Fernandes, Mallone Lopes Silva, Diandra Araújo Luz, Antônio Rafael Quadros Gomes, Marta Chagas Monteiro, Christiane Schineider Machado, Yohandra Reyes Torres, Tatiana Onofre de Lira, Antonio Gilberto Ferreira, Enéas Andrade Fontes-Júnior, Cristiane Socorro Ferraz Maia

**Affiliations:** ^1^Programa de Pós-Graduação em Ciências Farmacêuticas, Instituto de Ciências da Saúde, Universidade Federal do Pará, Belém, PA, Brazil; ^2^Programa de Pós-Graduação em Neurociências e Biologia Celular, Belém, PA, Brazil; ^3^Departamento de Química, Universidade Estadual do Centro-Oeste (UNICENTRO), 85010-990 Guarapuava, PR, Brazil; ^4^Departamento de Química, Universidade Federal de São Carlos (UFSCar), 13565-905 São Carlos, SP, Brazil; ^5^Laboratório de Farmacologia da Inflamação e do Comportamento, Faculdade de Farmácia, Instituto de Ciências da Saúde, Universidade Federal do Pará, 66075-900 Belém, PA, Brazil

## Abstract

Propolis is a resin produced by bees from raw material collected from plants, salivary secretions, and beeswax. New therapeutic properties for the Central Nervous System have emerged. We explored the neurobehavioral and antioxidant effects of an ethanolic extract of yellow propolis (EEYP) rich in triterpenoids, primarily lupeol and *β*-amyrin. Male Wistar rats, 3 months old, were intraperitoneally treated with Tween 5% (control), EEYP (1, 3, 10, and 30 mg/kg), or diazepam, fluoxetine, and caffeine (positive controls) 30 min before the assays. Animals were submitted to open field, elevated plus maze, forced swimming, and inhibitory avoidance tests. After behavioral tasks, blood samples were collected through intracardiac pathway, to evaluate the oxidative balance. The results obtained in the open field and in the elevated plus maze assay showed spontaneous locomotion preserved and anxiolytic-like activity. In the forced swimming test, EEYP demonstrated antidepressant-like activity. In the inhibitory avoidance test, EEYP showed mnemonic activity at 30 mg/kg. In the evaluation of oxidative biochemistry, the extract reduced the production of nitric oxide and malondialdehyde without changing level of total antioxidant, catalase, and superoxide dismutase, induced by behavioral stress. Our results highlight that EEYP emerges as a promising anxiolytic, antidepressant, mnemonic, and antioxidant natural product.

## 1. Introduction

Propolis is a bee resinous product elaborated from different parts of plants such as buds, bark, and tree exudates by bees. The chemical composition of propolis is highly variable and depends directly on the geographic origin and local flora. In addition, the season, climate, bee species or genus, and extraction method also influence the composition of the final extract of propolis [1–5].

Pharmacological effects have been described for propolis related to their different constituents. Regarding the Central Nervous System (CNS), new therapeutical tools have emerged. In fact, since 2003, bioactive components present in propolis have been investigated. For example, caffeic acid phenethyl ester (CAPE) studies have claimed this compound as a neuroprotector in a cerebral inflammatory model [6]. After that, CAPE demonstrated its effectiveness on models of neuroinflammation, such as the cerebral ischemia model [7], and glutamate-induced excitotoxicity [8], through antioxidant or p38 phosphorylation and caspase-3 activation, respectively. In addition, the flavonoid pinocembrin, an important propolis constituent, has reduced brain lesion in an ischemia-reperfusion model, probably by its antioxidant and antiapoptotic activity [9].

In addition to flavonoids, other classes of compounds have been identified in several types of Brazilian propolis (green, red, and brown propolis) such as prenylated *p*-coumaric acids, acetophenone derivatives, lignans, other phenolic compounds, and di- and triterpenes [2]. Several samples of Brazilian yellow propolis were previously grouped according to their physicochemical and biological properties [10]; however, Brazilian yellow propolis has been scarcely studied [11]. Similar to yellow propolis from Cuba, sample of yellow propolis collected in the Central-Western Region of Brazil was mainly composed by triterpenoids belonging to oleanane, lupane, ursane, and lanostane skeletons [11–13].

Extracts of propolis have demonstrated several activities on the CNS. For example, the natural extract attenuated seizures induced by kainic acid, at least in part, via adenosine A1 receptor modulation [14]. In addition, a study [15] concluded that propolis elicited neuroprotected effect when given in combination with an anticonvulsant drug (e.g., valproate), against the neurophysiological disorders induced by pilocarpine epilepsy in rats, restoring the hippocampal neurotransmitter levels (e.g., dopamine and serotonin).

Nevertheless, solely in 2012, the effectiveness of propolis was investigated in psychiatric disorders. Firstly, a study demonstrated that the essential oil of Brazilian green propolis, rich in terpenoids, produces therapeutic effects on anxiety by hyperfunction of hypothalamic-pituitary-adrenal (HPA) axis reduction [16]. Researchers reported [17] that ethanol extract of Korean propolis exerted antidepressant activity by enhancing glucocorticoid receptor (GR) function. In this sense, our group hypothesized that the oil extract of brown propolis collected in south Brazil could potentiate the behavioral effects demonstrated previously by Lee experiment [17]. In fact, the oil extract of this brown propolis exerted stimulant, anxiolytic, and antidepressant effects at lower doses [18].

In the present paper, we explored the neurobehavioral effects of the ethanolic extract of yellow propolis sample collected in Mato Grosso do Sul, Brazil, which was demonstrated to be rich in triterpenoids [11]. Triterpenoid compounds have shown sedative, tranquillizer, and anticonvulsant activities [19–22].

## 2. Material and Methods

### 2.1. Propolis Origin, Extract Preparation, and Chromatography Fractionation

#### 2.1.1. Propolis Sample

The yellow propolis sample produced by* Apis mellifera* bees was collected in January 2012 in Mato Grosso do Sul, Brazil. Yellow propolis was stored at −18°C until extraction [11].

#### 2.1.2. Propolis Extraction

Yellow propolis sample was ground with a mortar and pestle and extracted by maceration in AP ethanol (Biotec, Brazil) (1 : 10 w/v) for 24 hours using an incubator (Tecnal, Brazil) at 160 rpm and room temperature. After the extraction time, the solution was vacuum-filtered to remove the insoluble parts. Then, the hydroalcoholic phase was placed in a freezer for 24 hours. After this time, the solution was again filtered through qualitative filter paper (Macherey-Nagel, Germany) to remove waxes. The extraction solution was dried by evaporating the solvent under reduced pressure rotary evaporator (Model 752, Fisatom, Brazil), equipped with vacuum pump (Prismatec, Brazil) and a water bath (Nova Química, Brazil) at 50°C. The dry ethanolic extract of the yellow propolis (EEYP) was weighed (yield 19.80% w/w) and a sample was solubilized with Tween 20 solution 5% using vortex apparatus for 30 minutes to a final concentration of 5 mg/mL.

#### 2.1.3. Chromatography Fractionation

Three hundred milligrams of EEYP in hexane solution was applied on a silica gel chromatography column (particle size: 0.063–0.200 mm; pore size: 60 Å, MERCK G60; column size 3 × 80 cm). Elution was carried out with mixtures of increasing polarity organic solvents (hexane (100%), hexane/ethyl acetate (50 : 50, v/v), ethyl acetate (100%), and ethyl acetate/methanol (50 : 50, v/v)) to afford five major subfractions (F1 = 123.7 mg, F2 = 93.0 mg, F3 = 110.7 mg, F4 = 54.1 mg, and F5 = 84.1 mg).

### 2.2. Drugs and Solutions

The positive control treatments included diazepam (DZP: 7-chloro-1-methyl-5-phenyl-1,3-dihydro-2 H-1,4-benzodiazepin-2-one, Diazepamil®, Hipolabor Laboratory, Brazil), fluoxetine (FXT: N-methyl-3-phenyl-3-[4-(trifluoromethyl) phenoxy] propan-1-amine hydrochloride, Fluxene®, Eurofarma Laboratory, Brazil), and caffeine (CAF: Sigma-Aldrich®, USA). Tween 80 solution (5%) was used (Sigma-Aldrich, USA, distilled water and sodium chloride).

### 2.3. Animals

Three-month-old male Wistar rats (body weight = 122 ± 0.05 g; *n* = 10 per group) obtained from the Animal Facility, Biological Sciences Institute, Federal University of Pará (UFPA), were used in the experimental assays and kept in collective cages (5 animals per cage). Animals were maintained in a climate-controlled room on a 12 h reverse light/dark cycle (lights on 7:00 AM), with food and water* ad libitum*. All procedures were approved by the Ethics Committee on Experimental Animals of the Federal University of Pará under license number BIO-046-12 and followed the guidelines suggested by the NIH* Guide for the Care and Use of Laboratory Animals*. All behavioral assays were performed in the Laboratory of Pharmacology of Inflammation and Behavior at the Federal University of Pará.

Animals were divided into eight groups, defined as control group (vehicle) that received saline solution plus Tween 80 (5%) intraperitoneally (i.p.); EEYP at 1, 3, and 30 mg/kg i.p.; and positive control group diazepam 1 mg/kg i.p., for anxiety-like tests; fluoxetine 10 mg/kg i.p., for antidepressant-like test; and caffeine 10 mg/kg i.p., for memory test.

### 2.4. Behavioral Assays

#### 2.4.1. Open Field (OF) Test

EEYP, saline solution plus Tween 80 (5%), or diazepam was administered 30 min before the behavioral tests. Rats were placed individually in the center of a wooden arena (100 × 100 × 40 cm) divided into 25 quadrants to evaluate the number of sections visited by the animal over a period of 5 min. The test was videotaped and analyzed by Any Maze Stoelting software (USA). The parameters of total distance traveled (motor index), time spent, and distance traveled in the central area (emotional index) were measured.

#### 2.4.2. Elevated Plus Maze (EPM) Test

Following the open field (OF) test, the animals were subjected to the elevated plus maze (EPM) test, which consists of a plus-shaped wooden maze with two opposite open arms (50 × 10 cm) and two enclosed arms (50 × 10 × 40 cm) spreading out from a central platform (10 × 10 cm) elevated at a height of 50 cm from the floor. The animals were individually placed in the center of the EPM, facing one of the enclosed arms, and were allowed to explore the apparatus for 5 min following the previously described protocol [23]. The parameters measured were frequency of open arm entries (OAE); open arm time (OAT); and frequency of enclosed arm entries (EAE). The % OAE and % OAT were calculated according to the formula [(open/total) × 100]. An entry was counted whenever the animal placed four paws in an arm of the maze. An anxiogenic effect is defined as a decrease in the % OAE and/or % OAT.

#### 2.4.3. Forced Swimming (FS) Test

Following the EPM test, the animals were subjected to the forced swimming (FS) test. Rodents were individually dropped into cylindrical tank (50 cm in diameter; 70 cm high) containing water at 23 ± 1°C and were monitored for 5 min in inescapable conditions. Immobility time was recorded during the last 3 min. The first 2 min were considered habituation. The rats were judged as immobile whenever they stopped swimming and floated in an upright position for 2 s and when only small movements to keep their head above the water level were observed. The reduction in the immobility time was considered an antidepressant-like effect. The number of climbing events was measured to evaluate motor skills [24].

#### 2.4.4. Step-Down Inhibitory Avoidance (IA) Test

Animals were submitted to the inhibitory avoidance (IA) apparatus that was an acrylic box (50 × 25 × 25 cm^3^) whose floor consisted of parallel stainless steel bars (1 mm in diameter) spaced 1 cm apart (Insight, Brazil). A platform (7 cm wide × 2.5 cm high) was placed on the floor against the left wall of the box. Using a previously reported protocol [25, 26], animals were placed on the platform and the time from latency to step-down on the grid with four paws was measured with an automatic device. Latency time was used as a measure of memory retention (maximum 180 s). A previous training session was carried out by giving the animals a 0.4 mA, 1.0 s scrambled foot shock immediately after they stepped down on the grid. Then, the animals were immediately removed from the apparatus until next session. In order to evaluate short-term memory, the test sessions were performed 1.5 h after training.

In addition to behavioral parameters already observed in the battery of tests, animals were also evaluated to detect the occurrence of adverse effects that may suggest toxicity. Signals of both stimulant (snout scratching, tremors, increased respiratory rate, paw licking, tail biting, arousal, nasal discharge, piloerection, stereotyped movements, and convulsions) and depressant (alienation of the environment, ataxia, catatonia, decreased respiratory rate, apathy, dyspnea, ptosis, sedation, and dorsal tone) activities were evaluated [27].

After the behavioral tests, blood samples were collected to determine oxidative stress.

### 2.5. Oxidative Biochemistry Assays

Experiments to evaluate oxidative stress were designed in the* In Vitro* Activities Laboratory at the Federal University of Pará. After the behavioral assays, animal's blood samples were obtained by intracardiac puncturing. A basal group that was not submitted to the behavioral protocol was added to the biochemical oxidative assay. Nitric oxide (NO), malondialdehyde (MDA), trolox equivalent antioxidant capacity (TEAC), catalase (CAT), and superoxide dismutase (SOD) levels were measured.

#### 2.5.1. Determination of Plasma Nitric Oxide (NO) Concentration

The nitrate (NO_3_^−^) present in the serum samples was converted to nitrite with nitrate reductase, and the nitrite concentration was determined using the Griess method [28]. Briefly, 100 *μ*L of the supernatant samples was incubated with an equal volume of Griess reagent for 10 min at room temperature. The absorbance was measured on a plate scanner (Spectra Max 250; Molecular Devices, Menlo Park, CA, USA) at 550 nm. The nitrite (NO_2_^−^) concentration was determined using a standard curve generated using sodium nitrite (NaNO_2_). Nitrite production was expressed per *μ*M.

#### 2.5.2. Determination of Plasma Malondialdehyde (MDA) Concentration

The MDA was used for the reaction of thiobarbituric acid reactive substances (TBARS) performed according to the adapted form [29] of a previously proposed method [30]. An aliquot of 1 mL of the reagent (TBA 10 nM) and 0.5 mL of the sample were added to each test tube. Then, the tubes were placed in a water bath at 94°C for 1 h. After this procedure, the samples were cooled in running water for about 15 minutes and then 4 mL of butyl alcohol was added to each sample. Subsequently, the samples were mixed on a vortex shaker, in order to obtain the maximum extraction of MDA into the organic phase. Finally, the tubes were centrifuged at 2,500 rpm for 10 minutes. A volume of 3 mL of supernatant was pipetted to carry out spectrophotometric reading at 535 mm. Results were expressed in nmol/mL.

#### 2.5.3. Measurement of Trolox Equivalent Antioxidant Capacity (TEAC)

The trolox equivalent antioxidant capacity (TEAC) is a sensitive and reliable marker for detecting* in vivo* oxidative stress markers that may not be detectable through the measurement of a single, specific antioxidant [31]. TEAC level of the blood serum sample was measured using a previously developed method [32]. In this assay, 7 mM of 2,2-azinobis, 3-ethylbenzothiazoline, 6-sulfonate (ABTS) was incubated with 2.45 mM of potassium persulfate and the ABTS-potassium persulfate (1 : 0.5, v/v) and the mixture was allowed to stand in the dark at room temperature for 12–16 h before use. For the study, the blue-green ABTS+ solution was diluted with ethanol 95% (v/v) until the absorbance reached 0.70 ± 0.02 at 734 nm. Then, 10 *μ*L of the blood serum or trolox standard was mixed with 1 mL of ABTS+ solution, and decrease in absorbance at 734 nm was recorded after 4 min for all samples. The absorbance of the mixture was monitored at 734 nm after 6 min. For the blank, 10 *μ*L of water instead of the sample was used and each sample was measured in triplicate. Total antioxidant potential of blood serum was expressed as *μ*mol/mL of TEAC and was calculated through a calibration curve plotted with different amounts of trolox [33].

#### 2.5.4. Measurement of Catalase (CAT) Activity

CAT activity was determined according to a previously validated method [34]. Blood samples were haemolysed into ice water (1 : 3) and then diluted in a Tris based buffer (Tris 1 M/EDTA 5 mM, pH 8.0). To verify the decay of hydrogen peroxide (H_2_O_2_), aliquots of the diluted samples were added to 900 *μ*L of the reaction solution (Tris base, H_2_O_2_ 30% and ultrapure water, pH 8) [35]. The decrease of H_2_O_2_ concentration was established at *λ* = 240 nm at 25°C for 60 seconds. CAT activity was defined as the activity required to degrade 1 mol of H_2_O_2_ during 60 seconds (pH 8 and 25°C) and was expressed as U/mg protein. The molar extinction coefficient of H_2_O_2_ used for the calculation was 39.4 cm^2^/mole. The enzymatic activity data obtained in CAT assays were normalized by the total protein concentrations, using the commercial kit (Doles, Brazil).

#### 2.5.5. Measurement of Superoxide Dismutase (SOD) Activity

Determination of SOD activity was performed following reported recommendations [36]. For this, blood samples were haemolysed into ice water (1 : 3) and then diluted in a Tris based buffer (Tris 1 M/EDTA 5 mM, pH 8.0). Total SOD activity of blood sample was determined by the inhibition of cytochrome c reduction [37]. This method evaluated the ability of SOD to catalyze the conversion of superoxide anion (O_2_^−^) to hydrogen peroxide (H_2_O_2_) and oxygen gas (O_2_). The reduction of cytochrome c was mediated by superoxide anions generated by the xanthine/xanthine oxidase system and monitored at a wavelength of 550 nm. One unit of SOD was defined as the amount of enzyme required to inhibit the rate of cytochrome c reduction by 50%. SOD activity was measured using ultraviolet-visible (UV-VIS) spectrophotometer at a wavelength of 550 nm and was expressed in nmol/mL.

### 2.6. ^1^H-NMR Analysis of EEYP and Fractions

Proton nuclear magnetic resonance (^1^H-NMR) analysis was recorded at 298 K in a Bruker UltraShielding*™* Plus 600 MHz spectrometer operating at 14.6 T. EEYP and fractions were dissolved in a mixture of 100 *μ*L D_2_O (buffer phosphate, pH 7.04) and 600 *μ*L CD_3_OD. The solution was centrifuged at 13,000 rpm for 20 min at room temperature. The supernatant (600 *μ*L) was transferred into an NMR tube of 5 mm. ^1^H-NMR spectra were acquired using a NOESY pulse sequence for presaturation on water resonance and spoiled gradient during mixing time (noesygppr1d, Bruker terminology). The parameters settled in this sequence were 4.0 s for relaxation delay time, acquisition time of 3.99, data points of 140 k, mixing time of 10 ms, and 128 scans with a spectral window of 30 ppm. Spectra were processed by applying an exponential line broadening LB of 0.3 and manually phased trough Topspin 3.0 (Bruker Biospin).

### 2.7. Statistical Analysis

Data were analyzed using one-way analysis of variance (ANOVA) followed by Tukey's test for multiple comparisons of behavior and oxidative stress test results. The Student* t-*test was used for multiple comparisons in determining NO. Data from each experimental group were expressed as the mean ± standard error of the mean (SEM) of 10 animals per group. *P* values less than 0.05 (*P* < 0.05) were considered to be indicative of significance. The graphical construction and statistical analysis were performed using GraphPad Prism 5.0 software (San Diego, California, USA).

## 3. Results

### 3.1. Yellow Propolis Did Not Cause Toxic or Adverse Effects During Tests

EEYP did not elicit signals of toxicity reactions or adverse reactions. In fact, animals did not present toxic symptoms at the beginning (30 min after EEYP administration) nor at the end of the behavioral tests (195 min after EEYP administration).

### 3.2. Yellow Propolis Does Not Change the Animals Deambulation in the OF and It Exacerbates the Time of Exploration of Central Squares

**Figure 1:**
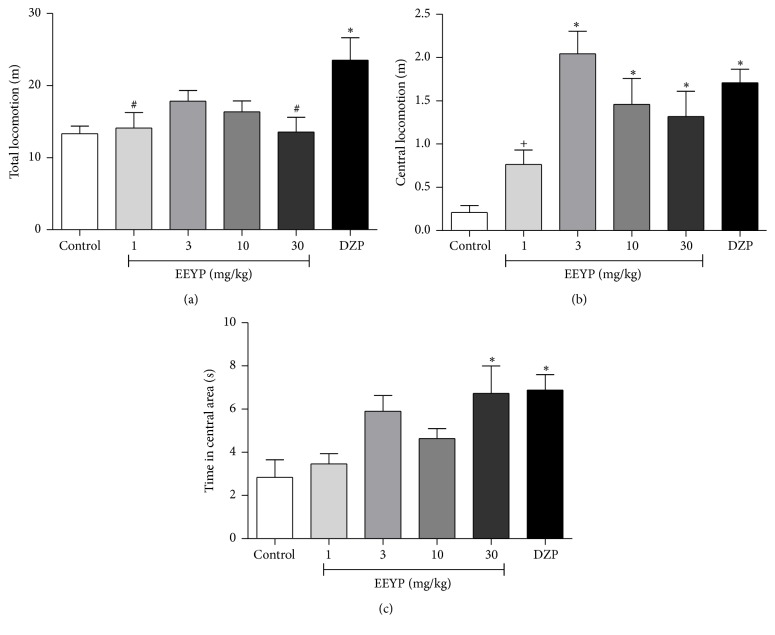
Effect of treatment with EEYP (1, 3, 10, and 30 mg/kg) on (a) total locomotion; (b) time in central area; and (c) central locomotion in the open field test. ^*∗*^ *P* < 0.05* versus* control group; ^#^ *P* < 0.05* versus* diazepam (DZP) group; ^+^ *P* < 0.05* versus* EEYP 3 mg/kg group. Values are expressed as mean ± SEM from 10 animals per group (ANOVA and Tukey's test).

EEYP did not interfere in the animal ambulation, indicating no sedative effect of propolis (Figure 1(a)).

Figure 1(b) shows the results of locomotion in the central squares which is a parameter related to anxiety-like behavior. It was observed that animals treated with EEYP at dose ≥3 mg/kg had ambulation in the central area increased. Besides, administration of EEYP (30 mg/kg) also increased the exploration time spent in the central quadrants (Figure 1(c)), which suggests an anxiolytic-like effect.

### 3.3. Yellow Propolis Promotes Anxiolytic-Like Behavior in the EPM Test

**Figure 2:**
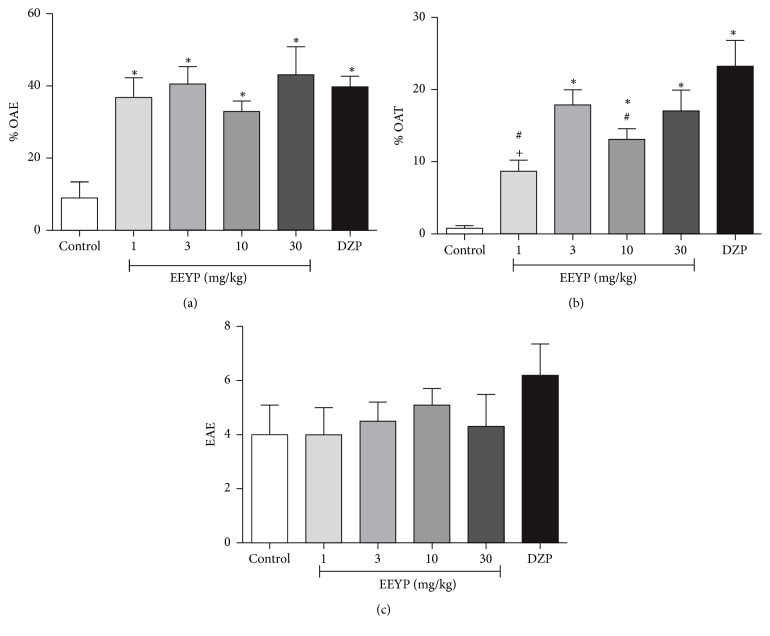
Effect of treatment with EEYP (1, 3, 10, and 30 mg/kg) on (a) entrances in the open arms (%); (b) time in the open arms (%); and (c) entrances in the enclosed arms in the elevated plus maze test. ^*∗*^ *P* < 0.05* versus* control group; ^#^ *P* < 0.05* versus* diazepam (DZP) group; ^+^ *P* < 0.05* versus* EEYP 3 mg/kg group. Values are expressed as mean ± SEM from 10 animals per group (ANOVA and Tukey's test).

In the EPM test, EEYP increased the % OAE (*P* < 0.05, Figure 2(a)). In addition, animals treated with EEYP showed significant increase in the % OAT parameter (Figure 2(b); *P* < 0.05), confirming previous anxiolytic-like behavior, observed in the OF test. Indeed, animals treated with EEYP showed a similar behavior as those treated with the standard anxiolytic drug diazepam, showing the effectiveness and potential of yellow propolis in anxiolytic therapy.

The EAE parameter was not altered in the animals treated with EEYP or with diazepam (Figure 2(c)), which shows that the animals did not present any level of sedation, which could impair motor behavior and consequently deambulation.

### 3.4. Yellow Propolis Promotes Antidepressant-Like Activities in FS Test

**Figure 3:**
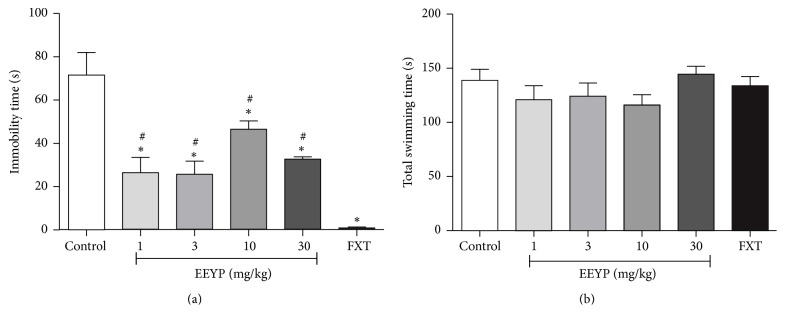
Effect of treatment with EEYP (1, 3, 10, and 30 mg/kg) on (a) immobility time and (b) total swimming time in the forced swim test. ^*∗*^ *P* < 0.05* versus* control group; ^#^ *P* < 0.05* versus* fluoxetine (FXT) group. Values are expressed as mean ± SEM from 10 animals per group (ANOVA and Tukey's test).

In the FS model, animals showed significant decrease in the immobility time (*P* < 0.05), indicating antidepressant-like behavior (Figure 3(a)). However, EEYP seems to be less effective than the standard drug fluoxetine (positive control).

The total swimming time (Figure 3(b)) of the animals treated with EEYP was not modified, which suggests that motor function was preserved. This result corroborates those observed in the OF and EPM tests, where the treated animals did not show motor behavior or spontaneous deambulation affected.

### 3.5. Yellow Propolis Promotes Cognitive Effects in the Step-Down IA Test

**Figure 4:**
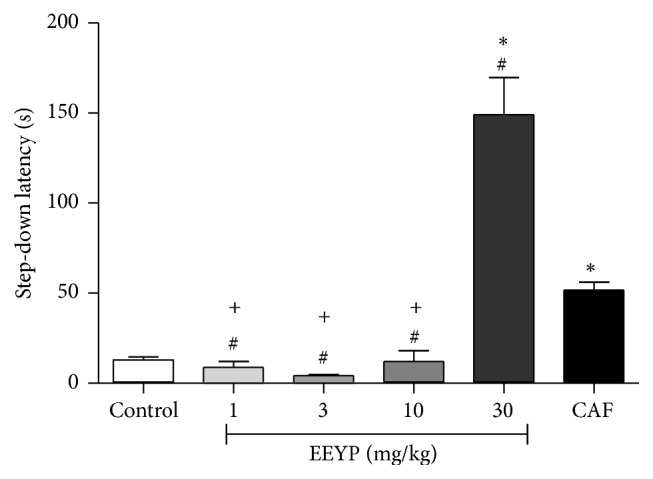
Effect of treatment with EEYP (1, 3, 10, and 30 mg/kg) on the step-down latency in the step-down inhibitory avoidance (IA) test. ^*∗*^ *P* < 0.05* versus* control group; ^#^ *P* < 0.05* versus* caffeine (CAF) group; ^+^ *P* < 0.05* versus* EEYP 30 mg/kg group. Values are expressed as mean ± SEM from 10 animals per group (ANOVA and Tukey's test).

Animals treated with EEYP at 30 mg/kg showed an increase in the step-down latency, which indicates positive mnemonic activity at this dose (Figure 4). Surprisingly, such effect was more prominent than caffeine group (positive control group).

### 3.6. Yellow Propolis Shows Antioxidant Activity in the Oxidative Stress Evaluation

**Figure 5:**
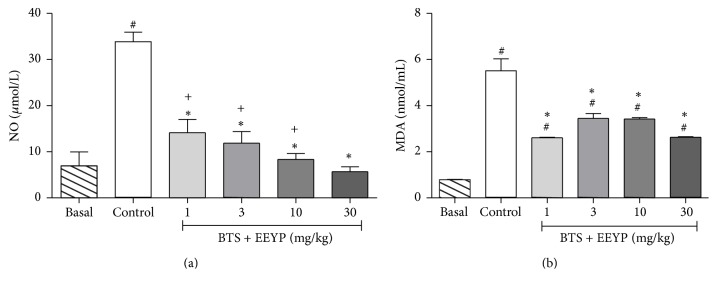
Effect of treatment with EEYP (1, 3, 10, and 30 mg/kg) on (a) nitric oxide (NO) and (b) malondialdehyde (MDA) levels of rats subjected to behavioral training stress (BTS). ^*∗*^ *P* < 0.05* versus* control group; ^#^ *P* < 0.05* versus* basal group; ^+^ *P* < 0.05* versus* EEYP 30 mg/kg group. Values are expressed as mean ± SEM from 10 animals per group (ANOVA and Tukey's test).

The behavioral training stress (BTS) induced by behavioral tests* per se*, represented by the control group, showed increased NO levels. Treatment with EEYP reversed this effect at dose-dependent manner, returning to basal levels even at the lowest dose 1 mg/kg (Figure 5(a)). In addition, the control group also exhibited a significant increase in serum MDA levels, which highlights the existence of oxidative stress in these animals compared to basal group. All doses of EEYP were able to inhibit the lipidic peroxidation process, decreasing MDA levels compared to control group, which may indicate that EEYP exhibits antioxidant activity (Figure 5(b)).

**Figure 6:**
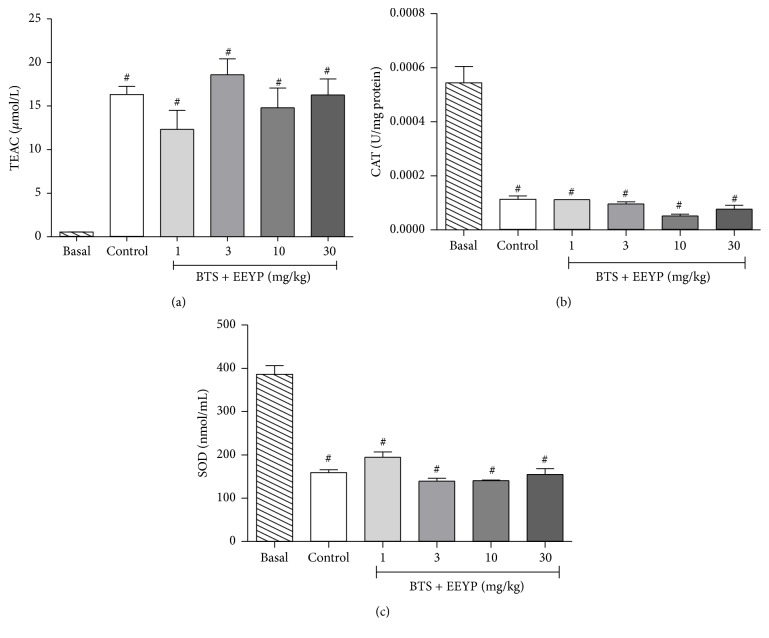
Effect of treatment with EEYP (1, 3, 10, and 30 mg/kg) on (a) trolox equivalent antioxidant capacity, TEAC; (b) catalase, CAT; and (c) superoxide dismutase, SOD, activity of rats subjected to behavioral training stress (BTS). ^*∗*^ *P* < 0.05* versus* control group; ^#^ *P* < 0.05* versus* basal group. Values are expressed as mean ± SEM from 10 animals per group (ANOVA and Tukey's test).

Regarding the antioxidant activity, behavioral tests increased TEAC levels and the treatment with EEYP did not change this parameter (Figure 6(a)). On the other hand, BTS inhibited antioxidant enzymes activities, such as CAT (Figure 6(b)) and SOD activities (Figure 6(c)). The treatment with EEYP also did not alter this enzymatic inhibition induced by behavioral stress, which indicates that others enzymatic or nonenzymatic pathways might be involved in antioxidant activity of EEYP. The ratio of TEAC to MDA was significantly higher in the EEYP-treated group if compared to control group (Table 1), mainly at dose of 30 mg/kg, confirming the antioxidant activity of EEYP.

### 3.7. Chemical Composition of Yellow Propolis

**Figure 7:**
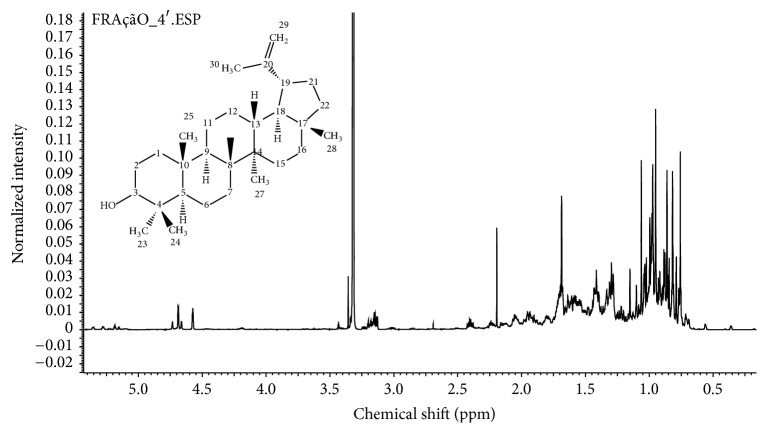
^1^H-NMR (600 MHz, D_2_O + CD_3_OD) of a chromatographic fraction (F4) containing lupeol as a main triterpene in yellow propolis.

Previously, we reported the occurrence of a mixture of triterpenes belonging to ursane, lupane, oleanane, lanostane, and cycloartane skeletons in the Brazilian yellow propolis sample from Mato Grosso do Sul under evaluation [11]. The presence of such lipophilic compounds and the absence of aromatic compounds were confirmed by ^1^H-NMR analysis of EEYP and fractions obtained by chromatography. By gas chromatography-mass spectrometry (GC-MS) analysis, lupeol and *β*-amyrin were identified as the main triterpenes in yellow propolis [11], representing 44.80% and 13.64%, respectively, of the total composition of the extract (see Table 1S in Supplementary Material available online at http://dx.doi.org/10.1155/2016/2906953). All fractions obtained by chromatographic fractionation of EEYP had similar chemical profiles by ^1^H-NMR and showed the lack of aromatic compounds and predominance of high field resonances. By ^1^H-NMR analysis of all fractions, lupeol was confirmed as one of the main constituents of yellow propolis (Figure 7) and its structure was assigned by comparing our data with ^1^H-NMR from literature (Table 2S in Supplementary Material). Intense singlets in *δ* 0.76, 0.82, 0.86, 0.95, 0.97, and 1.06 are consistent with methyl groups bonded to quaternary* sp* ^3^ carbons. The olefinic unit was confirmed by signals at *δ* 4.69 (doublet) and *δ* 4.58 (multiplet), indicating geminal olefinic hydrogens. A doublet of doublets at *δ* 3.14 is typical for hydrogens bonded to carbinolic carbons in triterpenes and the constant coupling of 11.19 Hz indicated axial to axial coupling (Figure 7).

## 4. Discussion

The present study demonstrates, for the first time, that a sample of Brazilian yellow propolis, which is rich in triterpenes, mainly lupeol, shows anxiolytic- and antidepressant-like activities. Triterpenes may promote behavioral effects related to antioxidant mechanisms in rats. Acute administration of EEYP starting at 1 mg/Kg induced anxiolytic- and antidepressant-like activities, as well as cognitive and antioxidant effects as could be observed by carrying out different behavioral tests (OF, EPM, FS, and IA) followed by biochemical analysis.

Several studies have demonstrated the therapeutic potential of propolis, particularly its widely established antioxidant action, which has been related to the presence of flavonoids and phenolic acids in its composition. The activity of propolis on the CNS has also been reported [38]. However, little is known about the properties of the yellow Brazilian propolis, which has low content of phenolic compounds but is rich in terpene compounds [11]. Actually, the presence of triterpenes gives lipophilic characteristics to this sample of yellow propolis and it may facilitate drug passages across the blood-brain barrier.

Plants containing terpenoid compounds have been used as sedatives, tranquillizers, and anticonvulsants. Many volatile oils rich in terpenes have a variety of pharmacological activities such as anxiolytic, anticonvulsant, and antinociceptive [21, 39, 40]. Actually, the presence of triterpenes provides lipophilic characteristics to the yellow propolis, which may facilitate passage across the blood-brain barrier [41].

The results obtained in the OF test allow us to infer that the locomotor activity remained preserved after administration of EEYP. Indeed, we can ensure that sedative effect and motor impairment induced by EEYP were excluded from the data obtained in the behavioral experiments. In addition, the OF test indicates that EEYP presented anxiolytic activity. Similar results were also reported in a study where an oily extract of greenish brown propolis (dose ≥ 10 mg/kg) was found to have similar anxiolytic effects in the central ambulation of treated rats [18].

The results obtained in the EPM test suggest that EEYP displays an anxiolytic-like effect starting at dose of 3 mg/kg that confirms the results obtained in the OF test. In addition, the walking ability of the animals was preserved in all treated groups, which indicates EEYP's ability to reduce anxiety while sedation was not induced. Similar results were obtained in animals treated with the oily extract of brown propolis at dose of 10 mg/kg [18]. As EEYP promoted such activity starting at 3 mg/Kg, we could infer that the hydroalcoholic extract was more potent than the oily extract [18], because it has elicited an anxiolytic-like effect in a dose at least 3-fold lower.

The behavioral results of animals treated with EEYP are consistent with studies of terpene compounds, such as carvacrol [42], (+)-epoxy limonene [43], linalool [44], 1,4-cineol [45], carvacryl acetate [46], and phytol [47], which also have shown anxiolytic action without changing locomotor activity.

In addition, our results are in agreement with a previous study [16], which found that the essential oil of a Brazilian green propolis sample, also rich in terpenes, reversed the anxiogenic behavior in mice and had no effect on locomotor activity. These effects were accompanied by a reduction in plasma levels of cortisol (CORT), adrenocorticotropic hormone (ACTH), and MDA with increase in SOD enzyme activity. These findings may suggest that the anxiolytic effects occur through the antagonism of hyperactivity of the HPA axis and the stimulation of antioxidant capacity in brain tissue [16].

Furthermore, several reports indicate that triterpenes exhibit anxiolytic-like activity. In this sense, a solution containing betulin, a triterpene structurally related to lupeol, has been patented as an anxiolytic remedy [48]. Posteriorly, it was demonstrated that betulin binds to gamma-aminobutyric acid A (GABA_A_) which could explain the anxiolytic and anticonvulsant properties of this compound [49]. However, the authors also observed that lupane derivatives, such as lupeol, were not able to bind to GABA_A_ receptors. In this sense, if lupeol produces anxiolytic effects, the probable mechanism does not include GABA_A_ stimulation pathway. In fact, it has been shown that an isomeric mixture of triterpenes alpha- and beta-amyrin (2.5 and 5 mg/Kg, i.p.) from crude resin of* Protium heptaphyllum* induces enhancement of noradrenergic mechanisms [50]. Hence, we suggest that the anxiolytic effect observed can be induced by the monoaminergic system [51]. However, complementary studies are necessary to clarify the compounds responsible for anxiolytic activity and the precise mechanisms of action.

Beyond the anxiolytic-like effects, the EEYP showed antidepressant-like activity in the FS test. A previous study [18] also observed such activity. However, our data demonstrated that the EEYP revealed more prominent effects, at least 10-fold higher than the oily extract of propolis [18]. Similar results were also found when investigating the ethanolic extract of Korean propolis sample [17], which revealed a dose-dependent antidepressant activity, without changes to motor function. Lee and coauthors have found a reduction in GR function in the hippocampus, as well as HPA axis after the FS test, which was reversed by the propolis extract that is considered as one of the mechanisms of antidepressant therapy and may explain one probable mechanism of the propolis antidepressant activity.

Additionally, the isomeric mixture of triterpenes has also exhibited antidepressant response by noradrenergic mechanisms [50]. Similar effects, however, involving serotonergic transmission were observed with monoterpenes [52]. Thus, it is possible that the triterpenes found in EEYP have elicited the antidepressant effect through the monoaminergic pathway. Nevertheless, further investigations to elucidate the antidepressant mechanisms are required.

In the step-down IA test, animals treated with EEYP at 30 mg/kg showed increase in the step-down latency, indicating mnemonic activity at this dose. Cognitive effects were also demonstrated by scopolamine-induced amnesia attenuation in rats after treatment with a water soluble fraction of propolis at dose of 100 mg/kg [53]. Furthermore, the fraction inhibited acetylcholinesterase activity in hippocampus of rats treated with scopolamine, enhancing the memory profile. In that study, the exact compound responsible for such effect was not identified; however, aromatic carboxylic acids and flavonoids were identified [53]. In this sense, our work highlights that the EEYP, which contains low levels of aromatic compounds and high contents of terpenes, triggered memory function at lower doses (30 mg/kg).

In stress conditions, excess generation of reactive oxygen species (ROS) and reactive nitrogen species (RNS) may lead to damage of various cell components, including induction of mitochondrial dysfunction, cytotoxic process of lipid cell membrane peroxidation, suppression of hippocampal synaptic plasticity [54], and triggering the activation of specific signaling pathways, such as proapoptotic pathways that result in apoptotic cell death [55]. Therefore, among other factors, such as genetic, neurochemical, neurobiological, and psychological, the oxidative stress plays an important role in the pathophysiology of anxiety and depression disorders [56]. In this regard, this oxidative/nitrosative stress can lead to the activation of intracellular signaling pathways and high levels of oxygen consumption in the brain, mainly in the hippocampus and prefrontal cortex, which consist of brain regions related to the pathophysiology of depression [56, 57]. Furthermore, oxidative stress also is associated with a dysregulation (hyper- or hypoactivity) of the hypothalamic-pituitary-adrenocortical (HPA) axis, which results in the increased levels of glucocorticoids (GCs) that alter antioxidant enzyme capacity, leading to the development of depressive disorders [58, 59]. During this oxidative process, increased lipid peroxidation and altered levels of antioxidant defenses, such as glutathione (GSH), CAT, and SOD enzymes, occur; therefore, strong antioxidants could be a promising approach to offering protection against anxiety and depression.

Regarding RNS, NO plays a vital role in CNS damage, since it reacts with the superoxide anion to produce peroxynitrite, which in turn triggers cell death by apoptosis process [60]. At least in part, the oxidative stress may elicit loss of the macromolecules function, as well as playing a key role in pathogenesis of several disorders, including neurodegenerative and others aging-associated diseases [61, 62]. Therefore, the TEAC activity increased altogether with higher NO and MDA levels in control group due to behavior stress, suggesting an enhanced ability to scavenge ROS in serum and tissue, as well as in the CNS. In this regard, our data also showed that EEYP treatment inhibited the NO levels elevation induced by BTS in a dose-dependent manner.

In the present study, lupeol was the main identified constituent of the yellow propolis. Lupeol acetate has been reported to significantly reduce NO production, inducible nitric oxide synthase (iNOS) protein levels, and lipopolysaccharide- (LPS-) induced cyclooxygenase- (COX-) 2 expression, showing antinociceptive and anti-inflammatory activities [63]. In another animal study, the lupeol also presented antinociceptive properties during inflammatory pain, inhibiting interleukin- (IL-) 1*β* and tumor necrosis factor- (TNF-) *α* production induced by carrageenan [64]. Recently, it was reported that the chloroformic fraction of* Lafoensia pacari*, rich in *β*-sitosterol and lupeol, displays antidepressant-like activity by an action mechanism dependent on serotonergic and catecholaminergic systems, as well as increase of the hippocampal brain-derived neurotrophic factor (BDNF) protein concentration [65].

Other components presented in propolis also showed both neuroprotective and antioxidant effects. In fact, investigation of pinocembrin, a flavonoid present in high concentrations in some propolis, revealed an* in vivo* neuroprotective effect and also a reduction in NO production [9]. Furthermore, EEYP also reduced MDA levels induced by BTS at dose as low as 1 mg/kg, which confirms the antioxidant action of EEYP. This same effect was also observed when testing the essential oil of Brazilian green propolis but at a dose of 100 mg/kg [16]. Previous studies have shown that the essential oil of a sample of Brazilian propolis reduces plasma MDA levels, one biochemical marker of lipid peroxidation [66, 67].

On the other hand, cellular defense mechanisms against ROS-induced oxidative stress, involving enzymatic and/or nonenzymatic factors, play a key role in the ROS elimination and detoxification process of xenobiotic compounds [61, 68]. In this regard, our data showed that EEYP did not alter TEAC, as well as the inhibition of SOD and CAT activities induced by behavioral stress. However, the ratio between TEAC and MDA levels showed that the EEYP treatment displays antioxidants levels elevation relative to prooxidants, which supports the antioxidant effects of EEYP (Table 1). We suggest that the antioxidant activity of EEYP may be due to other enzymatic or nonenzymatic pathways, as glutathione (GSH). In this regard, there was a report that brown propolis extract from southeastern Brazil increases GSH levels in the skin of rats submitted to ultraviolet (UV) irradiation [69]. In agreement with these results, our group has revealed an antioxidant activity of an oily extract of brown propolis from the south of Brazil, related to NO levels reduction, with no effects on total antioxidant capacity changes in rats exposed to BTS [18]. Based on the literature, we suggest that the antioxidant activity of EEYP can be similar to a standard antioxidant (i.e., vitamin E), which has shown ability to inhibit the production of MDA and NO, as well as trigger the SOD activity in the same level of EEYP after administration in animal models [70–72].

The antioxidant action of Brazilian green propolis has been attributed to several components, such as flavonoids and terpenes [73]. In the same way, the antioxidant properties of EEYP are probably related to its terpenes constituents. Some studies have shown that terpene compounds are responsible for a strong antioxidant activity [74–78]. Monoterpenes, such as (−)-myrtenol, have shown an* in vitro* antioxidant activity, which prevents lipid peroxidation, as well as removing nitrite ion concentrations and hydroxyl radicals [79]. In addition, new (ent-)abietane-type diterpenoids and norditerpenoids were isolated from* Chloranthus sessilifolius* plants [80]. These authors showed that the former compound inhibited NO production in LPS-stimulated BV-2 microglial cells, which evinces the antineuroinflammatory and antioxidant activities of these diterpenoids. Besides, (ent-)abietenes have been suggested as therapeutic alternatives for the treatment of neurodegenerative and other aging-associated diseases [80]. Moreover, the triterpenoid saponin (21-O-angeloyltheasapogenol E3; ATS-E3) isolated from* Camellia sinensis* plant seeds inhibited phagocytic uptake, ROS generation, and NO production by suppression of protein kinase B (PKB), also known as AKT; I kappa B kinase (IKK); and factor nuclear kappa B (NF-*κ*B)-dependent inflammatory pathways in macrophage culture [78].

Several studies have shown that the supplementation with antioxidants, such as N-acetyl-cysteine (NAC), lipoic acid, tocopherol, resveratrol, and propolis, leads to a neuroprotective effect induced by glutamate excitotoxicity. In this context, elevated levels of extracellular glutamate may inhibit cystine uptake, which displays a marked decrease in intracellular GSH levels and stimulates the NO production even as it produces free radical, such as mitochondrial superoxide anion products, which promotes oxidative stress [60, 81–83]. In this respect, some constituents of propolis may exert neuroprotective effects via an antioxidant effects. Brazilian green propolis and its main constituents (chlorogenic acid, 3,4-di-O-caffeoylquinic acid, and 3,5-di-O-caffeoylquinic acid) were reported to inhibit lipid peroxidation in mouse forebrain homogenates and protect against the glutamate-induced cell damage by antioxidant activity [60]. In addition, Brazilian green propolis was shown to have neuroprotective effects in an* in vitro* model of neurotoxicity and to consist of a more potent inhibitor of H_2_O_2_-induced cell death, as well as to protect against the* in vivo* ischemic neuronal damage in a mice model of neuronal occlusion [84]. Recently, it was reported that the treatment with hydroalcoholic extract of red propolis (HERP) at dose of 10 mg/kg has anti-inflammatory action and exhibits neuroprotective properties, showing a significant improvement in the sciatic nerve injury (SNI) and increased number of myelinated fibres [85].

Although several drugs are available in the neurological disorders (i.e., anxiety, depression, and cognition) therapy, there is a lack of new drugs with better tolerability and efficacy. In this context, our results demonstrated that a sample of Brazilian yellow propolis appears as a potential drug for the treatment of CNS disorders.

## 5. Conclusions

In conclusion, our results indicate for the first time that Brazilian yellow propolis rich in triterpenes, mainly lupeol, elicits neurobehavioral effects such as anxiolytics and antidepressants at dose as low as 1 mg/kg. Indeed, EEYP also displays cognitive effects in Wistar rats at the highest dose of 30 mg/kg and it did not promote sedation. However, additional studies are needed to evaluate the signaling pathways that may be affected which can support the behavioral results reported in the present work.

## Supplementary Material

Supplementary Material reports GC – MS data obtained for a sample of yellow Brazilian propolis and 1H-NMR data of lupeol, the most abundant triterpene in this yellow propolis.

## Figures and Tables

**Table 1:** Ratios between antioxidant (TEAC) factor and MDA levels of animals submitted to BTS and EEYP-treated BTS animals.

Groups	TEAC/MDA	*P* value
Control (BTS)	2.70 ± 1.23	
BTS+EEYP (1 mg/Kg)	4.82 ± 2.53^*∗*^	*0.0284*
BTS+EEYP (3 mg/Kg)	4.98 ± 2.50^*∗*^	*0.0186*
BTS+EEYP (10 mg/Kg)	4.23 ± 2.09	*0.0614*
BTS+EEYP (30 mg/Kg)	5.46 ± 2.19^*∗*^	*0.0027*

^*∗*^ *P* < 0.05 versus control group.

## List of Abbreviations

ABTS: 2, 2-Azinobis, 3-ethylbenzothiazoline, 6-sulfonate
ACTH: Adrenocorticotropic hormone
ANOVA: One-way analysis of variance
ATS-E3: 21-O-Angeloyltheasapogenol E3
BDNF: Brain-derived neurotrophic factor
BTS: Behavioral training stress
CAF: Caffeine
CAPE: Caffeic acid phenethyl ester
CAT: Catalase
CNS: Central Nervous System
CORT: Cortisol
COX: Cyclooxygenase
DZP: Diazepam
EAE: Frequency of enclosed arm entries
EEYP: Ethanolic extract of yellow propolis
EPM: Elevated plus maze test
FS: Forced swimming test
FXT: Fluoxetine
GABA_A_: Gamma-aminobutyric acid A
GR: Glucocorticoid receptor
GSH: Glutathione
HERP: Hydroalcoholic extract of red propolis
HPA: Hypothalamic-pituitary-adrenal
IA: Step-down inhibitory avoidance
IKK: I kappa B kinase
IL: Interleukin
iNOS: Inducible nitric oxide synthase
LPS: Protein levels and lipopolysaccharide
MDA: Malondialdehyde
NAC: N-Acetyl-cysteine
NF-*κ*B: Nuclear factor kappa B
NO: Nitric oxide
OAE: Open arm entries
OAT: Open arm time
OF: Open field test
PKB: Protein kinase B
RNS: Reactive nitrogen species
ROS: Reactive oxygen species
SEM: Standard error of the mean
SNI: Sciatic nerve injury
SOD: Superoxide dismutase
TBARS: Thiobarbituric acid reactive substances
TEAC: Trolox equivalent antioxidant capacity
TNF: Tumor necrosis factor
UV: Ultraviolet.
