# Supplementary material for: Neurobehavioral and Antioxidant Effects of Ethanolic Extract of Yellow Propolis
Source: Oxid Med Cell Longev. 2016 Oct 16;2016:2906953. doi: 10.1155/2016/2906953 (PMC5086397; doi:10.1155/2016/2906953)
Supplement: Supplementary file 1 — Supplementary Material reports GC – MS data obtained for a sample of yellow Brazilian propolis and 1H-NMR data of lupeol, the most abundant triterpene in this yellow propolis. [file 2906953.f1.docx]

**Supplementary Material**

NEUROBEHAVIORAL AND ANTIOXIDANT EFFECTS OF ETHANOLIC EXTRACT OF YELLOW PROPOLIS

Cinthia Cristina Sousa de Menezes da Silveira^a^, Luanna Melo Pereira Fernandes^a,b^, Mallone Lopes da Silva^a^, Diandra Araújo Luz^a^, Antônio Rafael Quadro Gomes^a^, Marta Chagas Monteiro^a,b^, Christiane Schineider Machado^d^, Yohandra Reyes Torres^d^, Tatiana Onofre de Lira^e^, Antonio Gilberto Ferreira^e^, Enéas Andrade Fontes-Júnior^a,b,c^, Cristiane Socorro Ferraz Maia^a,b,c^*

**Tabel 1S – Relative percentage of triterpenes identified by GC – MS (electron ionization) in a yellow Brazilian propolis extract.**

| *t_R_*(min) | Compounds | Relative composition (%) |
| --- | --- | --- |
| 12.5 | Lanosterol | 3.41 |
| 12.6 | 9,19-Cyclolanost-7-en-3-ol | 1.39 |
| 13.5 | *β*- Amyrin | 13.64 |
| 13.8 | Lup-20(29)-en-3-ona | 11.79 |
| 14.0 | Lupeol | 44.80 |
| 14.9 | Olean-12-en-3 one | 6.35 |
| 15.0 | Olean-12-en-3-yl acetate | 2.97 |
| 15.1 | Olean-18-en-3-ol | 2.10 |
| 15.6 | 13,27-cycloursan-3-ol (3*β*, 13*β*,14*β*) acetate | 6.09 |
|  | Unidentified compounds | 6.73 |

**TABLE 2S –Comparison of ^1^H-NMR data of lupeol (δppm, m, *J* Hz*,* at 600 MHz, solvent D_2_O+CD_3_OD) in yellow propolis and ^1^H-NMR data from literature.**

| Position | ^1^H-NMR | ^1^H-NMR ^a^ | ^1^H-NMR ^b^ | ^1^H-NMR ^c^ | ^1^H-NMR^d^ |
| --- | --- | --- | --- | --- | --- |
| 1 |  |  |  |  |  |
| 2 |  |  |  |  |  |
| 3 | 3.14 (dd, *J*= 11.19; 4.55 Hz) | 3.18 (1H, dd) | 3.16 (1H, dd) | 3.18 (dd) |  |
| 4 |  |  |  |  |  |
| 5 |  |  |  |  |  |
| 6 |  |  |  |  |  |
| 7 |  |  |  |  |  |
| 8 |  |  |  |  |  |
| 9 |  |  |  |  |  |
| 10 |  |  |  |  |  |
| 11 |  |  |  |  |  |
| 12 |  |  |  |  |  |
| 13 | 1.90 (m) |  | 1.89 (1H, m) | 1.67 (t) | 1.68 (m) |
| 14 |  |  |  |  |  |
| 15 |  |  |  |  |  |
| 16 |  |  |  |  |  |
| 17 |  |  |  |  |  |
| 18 |  |  |  |  |  |
| 19 | 2.40 (m) | 2.38 (1H, m) | 2.35 (1H, m) | 2.39 (m) | 2.37 (m) |
| 20 |  |  |  |  |  |
| 21 |  |  |  |  |  |
| 22 |  |  |  |  |  |
| 23 | 0.76 (s) | 0.76 (3H, s) | 0.73 (3H, s) | 0.98 (s) |  |
| 24 | 0.82 (s) | 0.79 (3H, s) | 0.76 (3H, s) | 0.77 (s) |  |
| 25 | 0.86 (s) | 0.83 (3H, s) | 0.80 (3H, s) | 0.84 (s) | 0.85 (s) |
| 26 | 0.95 (s) | 0.94 (3H, s) | 0.91 (3H, s) | 1.04 (s) | 1.08 (s) |
| 27 | 0.97 (s) | 0.97 (3H, s) | 0.94 (3H, s) | 0.97 (s) | 0.97 (s) |
| 28 | 1.06 (s) |  | 1.01 (3H, s) | 0.79 (s) | 0.80 (s) |
| 29 | 4.58 (m); 4.69 (d) | 4.57; 4.68 (d) | 4.53; 4.65 (2H, s) | 4.58 (m); 4.69 (m) | 4.58 (s); 4.70 (s) |
| 30 | 1.70 (s) | 1.68 (3H, s) | 1.65 (3H, s) | 1.69 (s) | 1.70 (s) |

^a^ Data reported by Marinho 2008 (CDCl_3_; 500 MHz), ^b^ Maldaner 2005 (CDCl_3_; 400 MHz), ^c^ Reynolds et al., (1986) and ^d^ de Albuquerque et al., (2007).

Albuquerque, I.L., Alves, L.A., Lemos, T.L.G., Monte, F.J. Q. 2007. Ácido canárico (3, 4-*seco* derivado do lupano) em própolis do Ceará. Quím Nova, 30, 828-831.

Maldaner, G. 2005. Estudos dos metabólitos secundários de *Condalia Buxifolia* e *Scutia Buxifolia* e suas atividades antimicrobiana, Master Thesis, Universidade Federal de Santa Maria.

Marinho, R.O.S. 2008. Estudo fitoquimico da especie B*yrsonima sericea* e sua aplicacao em dermocosmética, Master Thesis, Faculdade de Farmácia da Universidade Federal do Rio de Janeiro, Ciências Farmacêuticas.

Reynolds, W.F., McLean, S., Poplawski, J., Enriquez, R.G., Escobar, L.I., Leon, I. 1986. Tetrahedron 42, 3419-3428.
